# Development of mitochondrial replacement therapy: A review

**DOI:** 10.1016/j.heliyon.2020.e04643

**Published:** 2020-09-14

**Authors:** Hitika Sharma, Drishtant Singh, Ankush Mahant, Satwinder Kaur Sohal, Anup Kumar Kesavan, Samiksha

**Affiliations:** a Department of Zoology, Khalsa College Amritsar, Punjab, 143005, India; b Department of Molecular Biology and Biochemistry, Guru Nanak Dev University, Amritsar, Punjab, 143005, India; c Central University of Himachal Pradesh, India; d Department of Zoology, Guru Nanak Dev University Amritsar, Punjab, 143005, India

**Keywords:** Genetics, Molecular biology, Stem cell research, Cancer research, Biological sciences, Mitochondrial replacement therapies, IVF, Mitochondrial disorders, Genetic anomalies

## Abstract

Mitochondrial replacement therapy (MRT) is a new form of reproductive invitro fertilization (IVF) which works on the principle of replacing a women's abnormal mitochondrial DNA (mt-DNA) with the donor's healthy one. MRT include different techniques like spindles transfer (ST), pronuclear transfer (PNT) or polar body transfer (PBT). Transmission of defective mitochondrial DNA to the next generation can also be prevented by using these approaches. The development of healthy baby free from genetic disorders and to terminate the lethal mitochondrial disorders are the chief motive of this technique. In aged individuals, through in vitro fertilization, MRT provides the substitution of defective cytoplasm with cured one to enhance the expectation of pregnancy rates. However, moral, social, and cultural objections have restricted its exploration. Therefore, this review summarizes the various methods involved in MRT, its global status, its exaggerated censure over the years which depicts a strong emphasis for social acceptance and clinical application in the world of medical science.

## 1 Introduction

Mitochondrial Replace Therapy also referred to as “Mitochondrial Donation technique” (Jose et al., 2017) pertains to the category of techniques in which the embryo possessing the nuclear DNA of the parents is subjected to the IVF procedure to have mitochondrial DNA of the donor female (Schaefer and Labude, 2017). In this era of advanced scientific technologies, MRT aims to treat the infecundity of the diseased females and provide them a suitable chance to have biologically related healthy offspring (Kaur and Nagpal, 2017). Mitochondrial disorders have life threatening effects on the progenies of the diseased parents in which the point mutations are comparatively few. There are a number of diseases associated with defective mitochondria which are also the significant reason for the failure of metabolically active body organs like the heart, lungs, kidney, brain, and muscles *etc.* (Amato et al., 2014; Graff et al., 2002; Zeviani and Carelli, 2003). A few mt disorders respond to treatment. MRT technique is primarily concerned with the removal the mutated mitochondrial DNA of the mother. It saves the progeny from the adverse consequences of the mitochondrial disorders by providing healthy mt-DNA to the child. Registered point mutations in mitochondrial DNA are relatively uncommon and linked with a wide range of mitochondrial diseases influencing the retina, brain, optic nerve, muscle, endocrine organs, heart and liver (Graff et al., 2002; Zeviani and Carelli, 2003).

Since the mitochondrial genome codes for hundreds of proteins, it remains difficult to depict how one or more mutations have such considerable physiological effects. In addition, mt-DNA deletions build up with the ageing process and may contribute to the bioenergetics inability of older muscle fibres and neurons, leading to sarcopenia and neurological disorders such as Parkinson's and Alzheimer's (Wallace, 2010).

## 2 Mitochondrial disorders

Defects in the metabolism, impair the normal genotypic and phenotypic expression in the person. It results from the mutation in mt-DNA causing the malfunction of the respiratory chains, called the mitochondrial disorders. There are numerous copies of mt-DNA in the cell which differentiates it from the nuclear DNA (nDNA). This multiplicity strongly influences the type of mutation in mt-DNA and expression of mt-disorders (Castro, 2016). The beginning and severity of mt-disorders are dependent upon the amount shared by defective mt-DNA to a certain specified level called “threshold level” (Craven and Turnbull, 2019). The problem with mt-DNA is that it is unstable in nature and any alteration in it cannot be easily located (Fulk, 2018). About 100 babies are born with severe mt-disorders in UK majority of which die in the infant stage (Tavare, 2012). Set et al. (2019) reported that mutations in mt-DNA are responsible for 10–15% of mitochondrial diseases. It should be noted that mitochondrial dysfunction results from a mutation in both mt-DNA and nDNA (Palacios-Gonzalez, 2016). Some of the diseases associated with mitochondrial dysfunction are given in Table1.

**Table 1:** The most frequent mitochondrial disorders, their mutations and symptoms associated with them.

S.No	Mitochondrial Disorder	Mutations	Symptoms	Genotypic heterogeniety	Prevalence Rate	Reference
1	Leigh Syndrome	m.8993T > C; 10158T > C; m.10191T > C	Myoclonus, seizures, lesions and loss of mental efficacy and detrimental mobility, difficulty in breathing, kidney problems	MT-ND1; ND2; MT-ND3; , ND4; MT-ND5; MT-ND6; MT-ATP6; MT-TK; NDUFA2; NDUFA4; NDUFA9; NDUFA10; NDUFA12; NDUFS1; NDUFS2; NDUFS3; NDUFS4; NDUFS7; NDUFS8; NDUFAF2; NDUFAF5; NDUFAF6; NDUFV1; SURF1; SLC19A3; PNPT1; IARS2; NARS; ECHS1; VPS13D; NAXE; FOXRED1 of complex-I	1/40,000	Gerards et al. (2015); Khan et al. (2015); Tai (2016)Lee et al. (2020)
2	Pearson Syndrome	Moslty large mt-DNA deletions sporadic in nature, 4978-bp deletion	Asymptomatic. In some cases show vision loss and perversion of optic nerve cell.	tRNA ^gly^ (5^'^ endpoint: nt 9991); tRNA^thr^ (5^'^ endpoint: nt 15888)	uncertain	Rotig et al. (1991); Davison et al. (2019); Pronman et al. (2019)
3	Myoclonic Epilepsy with Ragged Red Fibres (MERRF)	m.8344A > G; m.8356T > C; m.8363G > A m.5703G > A; m.3291T > C; m.4279A > G;	Myopathy, cerebellar Ataxia, dementia and Myoclonus.	tRNA(lys) gene (MT-TK); tRNA(Asn) gene (MT-TN); tRNA (Leu) gene (MT-TL1); tRNA (Ile) gene; tRNA (Phe) gene (MT-TF); tRNA (Pro) (MT-TP)	0.25/100,00 In UK 95% rate in young ones	Tai, 2016; Jarovsky et al. (2006); Fu et al., (2019); Finsterer and Zarrouk-Mahjoub (2017)
4	Leiber's Herediatry Optic Neuropathy (LHON)	m.3460G > A; m.11778G > A; m.14484T > C	Asymptomatic. In some cases show vision loss and perversion of optic nerve cell.	MT-ND1; MT-ND4; MT-ND4L; MT-ND6; MT-ND5; Cytochrome-b (MT-CYB); Cytochrome-c oxidase (MT-CO3); tRNA (threonine) (MT-TT); tRNA (Glutamic acid) (MT-TE)	1/30,0000	Tai (2016); Davison et al. (2019); Bahr et al. (2020)
5	Neurogenic Ataxia and Retinitis Pigmentosa (NARP)	Mt-8993T > G/C; c.1092+5G > A	Dementia, seizures, ataxia, sensory neuropathy and retinitis pigmentosa.	MT-ATP6; FLVCR1 gene	1/2500-7000	Bainbridge (2010); Claeys et al. (2016); Lemoine et al. (2017); Kuehlewein et al., (2019)
6	Chronic Progressive External Opthalamoparesis (CPEO)	3243A > G	Dysfunction of extraocular muscles, ptosis and ocular myopathy.	POLG1, POLG2, ANT1, Twinkle, RRM2B, DNA2, SPG7, OPA1	uncertain	Klopstock and Mancuso (2019)
7	Mitochondrial Encepahalopathy Lactic Acidosis and Strokes like episodes (MELAS)	m.3243A > G	Muscle pain, weakness, vision enigma, detrimental mobility and brain damage.	MT-TL1	16/100,000	Jarovsky et al. (2006); Tai (2016); Bhatia et al. (2019)

## 3 Techniques involved in MRT

Two elementary approaches by which MRT is performed are pronuclear transfer (PNT) and maternal spindle transfer (MST). However, the third one polar body genome transfer has also been introduced.

### 3.1 Pronuclear transfer technique

**Figure 1:**
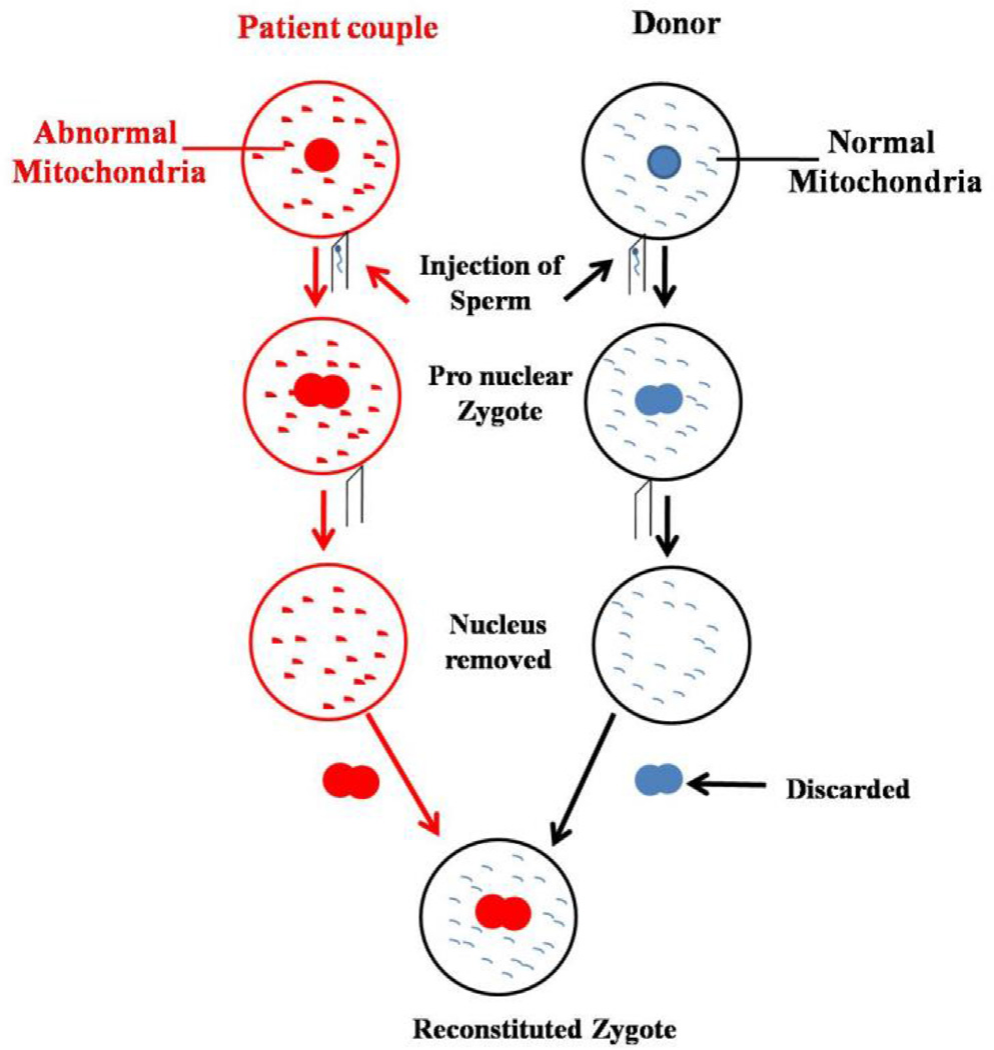
Steps involved in mitochondrial donation by Pronuclear Transfer Technique.

It is a significant approach of MRT administered after fertilization, in which two zygotes are raised in vitro (Figure 1). One of zygote belongs to the biological parents with pronuclei and defective mitochondria and the other one to the donor with pronuclei and healthy mitochondria (Kaur and Nagpal, 2017). The pronuclei of biological parents are taken out and transplanted into the donor's zygote (with rejected pronuclei) with healthy mitochondria by using electric pulses or inactivated hemagglutinating virus of Japan (Amato et al., 2014). The reformed zygote is transferred to the mother's womb. In general, the range of the carryover extends from being undetectable to less than 5% (Herbert and Turnbull, 2018). The technique trashes all the insecurities to grounds regarding its safety when the world's second three-parent baby was born in Ukraine with no clinical complications (Reardon, 2016).

### 3.2 Maternal spindle transfer (MST) technique

**Figure 2:**
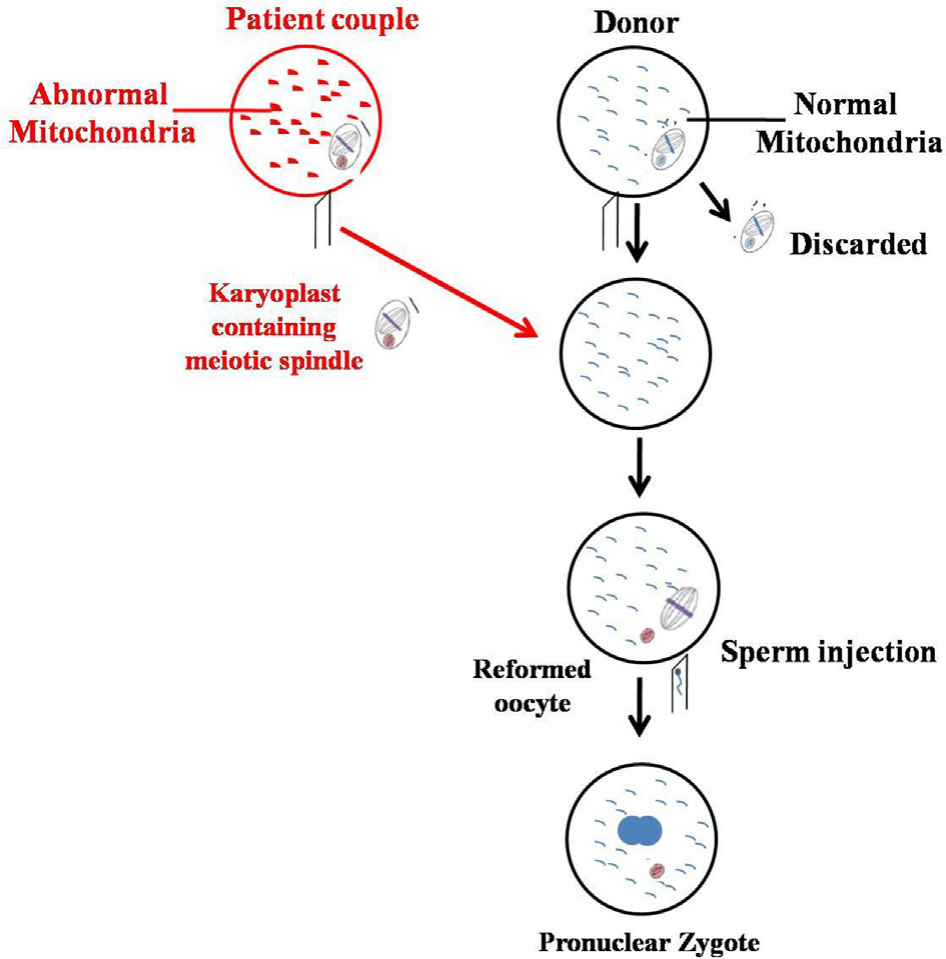
Steps involved in mitochondrial donation by Maternal Spindle Transfer Technique.

The technique, executed before fertilization is a form of selective reproduction similar to prenatal diagnosis and pre-implantation genetic diagnosis (PGD) (Wringley et al., 2015). The maternal spindle complex at the metaphase stage is extracted from the defective egg of the mother, which is then transplanted into the perivitelline space of the enucleated donor's egg with healthy mitochondria (Labarta et al., 2019) (Figure 2). The reformed embryo is transplanted into the mother's womb. This approach is preferable because maternal spindle contains little cytoplasm which eventually reduces the chances of mt-DNA carryover and mutations (Jose et al., 2017).

### 3.3 Polar body genome transfer (PBT)

Two small bodies with unequal cytoplasm and the equal number of the chromosomes as that of the primary oocyte's nucleus produced from the mammalian oocyte during the process of oogenesis are referred to as the polar bodies (BAC, 2018). The first polar body (PB1) is diploid in nature and second (PB2) is haploid (Wolf et al., 2015). It is considered as the most significant approach because of the presence of the scarce mitochondria with little cytoplasm which minimizes the possibilities of mt-DNA carryover. All the nuclear content enclosed within polar bodies increases its potency to confer the re-established oocytes and zygotes by the above two methods. The technique embraces standard micro-manipulation procedure with no usage of cytoskeleton disruptors minimizing the menace of any harm or injury to three individuals involved (BAC, 2018). The idea of the usage of the polar bodies was first put forth by Wakayama and Yanagimachi but adopted by Wang et al. (2014) to perform the technique in mice where, the transfer of first and the second polar body led to the normal progression of the progenies (Labarta et al., 2019).

## 4 Global stature of MRT

MRT has not been developed in the greater parts of the world till now (Garasic and Sperling, 2015). Very few countries have evoked their speculations by executing stringent inquiry, scientific opinions, and public debates. The studies on the efficacy and the risks associated with it are still under observation.

### 4.1 United Kingdom

MRT got successful approval from parliament of UK on February 2015 (Pompie and Pompie, 2018). It was the first country to make clear the legal existence of this method of reproductive intervention. Nuffield Council on Bioethics remarked the technique to be secured and applicable (Amato et al., 2014). The Human Fertilization and Embryology Authority (HFEA) also gave its no objection decision concluding that the matching of the mitochondrial haplotype and the donor's mitochondria is practically applicable (Chinnery et al., 2014). The authorization is based on several provisions including the clinical security of the manipulated gametes, the procedure being performed by experienced embryologists, and relevant counseling of the female diagnosed with mitochondrial disease (Herbert and Turnbull, 2018). In February 2017, HFEA granted the first license to the UK Fertility Clinic to start the first clinical trial that is still underway (Adashi et al., 2019).

### 4.2 United States

The United States opposed the adoption of a policy that makes it unjust. The argument put forward is that the executive privilege of the system is controlled by social and ethical peculiarities. Mitochondrial disorders with low causticity affect a small number of people, with a prevalence rate of 1/40,000 in America. Therefore, MRT cannot be prioritized to have excessive resources. The strategy is worthwhile because the hereditary kinship that is perplexed is reinforced (Rulli, 2016a, b). To date, 12,423 women in the US are at risk of transmitting mitochondrial diseases to their newborns (Adashi and Cohen, 2017). Several attempts to regulate MRT in the UK are actually urging FDA to reconsider its decision against MRT (Cohen et al., 2015).

### 4.3 Singapore

Following the United Kingdom, Singapore can legitimize the MRT technique. If the talks, aimed at the firmer inspection, to have successful outcomes, the country would be on the second position to legally approve the operation. Considerations are being made regarding the PBT approach to be implemented (Esson, 2018).

### 4.4 Australia

As reported, 20 children born per week are at a risk of developing traumatic mitochondrial diseases with one child per week having dreadful mitochondrial disorder (Han, 2018). The country is also heading for a detailed analysis of the disputed procedure and may also approve it, according to a Sydney-based newspaper (Aubusson, 2019). However, according to, Human Cloning for Reproduction Act, the practice is legally prohibited (Pritchard, 2018).

### 4.5 Mexico

The world's first, three-parent baby (boy) was born, showing no signs of genetic disorder on 6^th^ April 2015. MST has been adopted and successfully executed by Dr. John Zhang and his team (Hamzelou, 2016). The procedure was performed on a 36 year old Jordanian woman carrying 8993T > 9 mutations in subunit 6 of the ATPase gene which is the faulty mutation for Leigh Syndrome (Zhang et al., 2016). Leigh syndrome is not only due to variants in MT-ATP6, but more than 75 genes can be mutated. She suffered heteroplasmy with 23%, 24%, and 33% distorted mt-DNA in her blood, urine, and hair follicles respectively (Alikani et al., 2017) due to which she experienced four miscarriages and demise of two children (Pompie and Pompie, 2018). Actually ovarian stimulation cycles and oocyte manipulations were performed in New York and transplantation of the reformed embryo to the female's womb was accomplished at Affinity clinic in Guadalajara, Mexico, prior to which the consent was sought from Internal review board (IRB) of the Mexican clinic (Cohen, 2018).

### 4.6 Ukraine

The world's second three-parent baby was conceived by a 34 year old woman on 5^th^ January 2017. For the execution of the procedure, the permission was sought from the Ukrainian Association of Reproductive Medicine (Reardon, 2016). The procedure is not proscribed in the UK, so the technique was successfully operated by Dr. Valey Zukin, a fertility researcher, at Nadiya clinic in Kyiv, using PNT (Dockrill, 2017). The reports also affirmed that 7 babies have been born in Nadiya clinic so far, the most recent of which is the baby boy born on 18^th^ December 2018 (Walas, 2019).

### 4.7 Greece

Under successful clinical experiments, MRT made another noteworthy advancement in its field. A 32 year old Greek woman conceived a “three-parent baby” (boy) on 9th April, (2019) practicing the maternal spindle transfer technique by the two European fertility companies, Institute of life in Athens in collaboration with the Spanish Centre, Embryo tools (Kokkinidus, 2019). The child is labeled as the “Global's first” because the child was the product of the procedure treating infecundity, not a genetic disorder (Betuel, 2019).

## 5 Scope of MRT in India

MRT has not been explored much in India so far, but there are great possibilities for the success of this technique in the region. One in 5000 people suffers from mitochondrial impairment and the individual mutation rate is one in over 200 births, a rise in the last few years (Khan et al., 2015). In the first place, the perpetuation of descent and genetic connection with parents lays the basis for the role of the infant in society. Second, is the desire and social pressure to conceive a genetic child is highly prioritized (Knoppers et al., 2017). Due to this, the other reproductive alternatives like surrogacy and egg donation are not fully acceptable even today. It is agreeable that such norms are orthodox in today's world but the fact is that such norms exist and have formed deep roots in India which can't be eliminated easily. MRT fits into this arena fulfilling the hopes of diseased females aiming to give birth to the healthy child on their own.

## 6 Exaggerated criticism

The technique is to deal with the conflict between opponents and their underestimated complaints. The objections are exaggerated and short-sighted lacking profound scientific knowledge about the genetics of mt-DNA and the actual scientific approach behind the technique. Some of the objections to this technique are discussed.

### 6.1 Moral objections

The technique interferes with human decency by manipulating fragile embryos (Carr, 2015), while they have the same right to live (Dahiya and Garg, 2017). Ovarian stimulation is something of a personal activity, but it could be misused by others for business purposes (Dolitsky and Sauer, 2019). Alterations into natural systems should be prohibited. The identity of a person resides not only in his or her genes and traits, but also in his or her intimate relationship and social connections. The technique can disturb this relationship, impacting the cognitive and social well being of the newborn (Baylis, 2013).

### 6.2 Social objections

The technique is of low social value and is extremely expensive (Rulli, 2016a, b). Even if funded, economic issues for clinical implementation will be raised (Falk et al., 2016). A newborn may experience a confusing relationship with a donor who may be identified as a "second mother" or a "third parent." (Dimond and Stephens, 2017). According to Human Genetics Alert, this strategy is aimed to accomplish irresistible lineage preferences rather than child medical benefits (Armstrong, 2015). This is the product of a conservative ideology that prioritizes genetic links and rejects non genetic kinships (Schaefer and Labude, 2017). As a genetic contributor at the biological level, the donor may claim to be a parent in the future, making it a risky procedure (Douglas and Devolder, 2018).

### 6.3 Scientific objections

The main medical objection is that MRT is unable to cure mitochondrial disorders in patients or has developed in the human body (Rulli, 2016a, b). It also does not ensure prevention (Klopstock et al., 2016). While providing eggs to patients, the harm-benefit ratio is not sufficiently commendable for the continuation of the procedure (Palacios-Gonzalez, 2016). The procedure typically results in a wide variety of heteroplasm loads in newborns plodding up the effects (Gemmell and Wolff, 2015). Uncertainty in the capacity and inspection of the technique could injure three of the individuals involved (child, prospective mother, and donor) (Carr, 2015). Investing significant money and scarce resources to improve any medical treatment includes the seriousness and predominance of the disease in the population, which is not the case for mitochondrial disorders (Rulli, 2016a, b). The nuclear genome cooperates with mt-genome, which is important for the efficient development of energy in mitochondria (Chinnery et al., 2014). Any difference between them can lead to terrible medical outcomes (Reinhardt et al., 2013). It may have an effect on potential offspring, reasoned by Edward Morrow demonizing it as "Mito-nuclear Incompatibility" (Cavaliere and Gonzalez, 2018).

## 7 Significance of genetic empathy

Genetic empathy is the pillar of the child's identity and the universal standard for the protection of heritage, thereby maintaining a significant role in people's lives. Women are undeniably eligible to become mothers with genetically linked children (Gallapher, 2019). MRT is an evolutionary technique that can begin a new era by removing defective genome at zygotic and gametic level (Tachibiana et al., 2018).

### 7.1 Treatment of infecundity

Mitochondrial disorders destabilize the nuclear genome in mature oocytes, leading to embryo aneuploidy resulting in infection (Wolf et al., 2015). Owing to fear of passing dreadful diseases to their children, 78 percent of affected females were discouraged from having a child (Englestad et al., 2016). In this case, MRT is accessible. However, the reason behind the use of MRT to deliver three-parent babies in Ukraine was to treat the infection.

### 7.2 Conceptive autonomy

It is supposed to be the basic right of couples when and how to conceive their child. Whether or not they want a genetic child, or whether or not they want it through adoption or gamete donation, the decision should be theirs alone. No government or religious organization should be given the right to take moral decisions on behalf of couples and those suffering from it (Cavaliere and Gonzalez, 2018). It would be unfair to females with mitochondrial disease to limit their conceptive range (Schaefer and Labude, 2017). Apart from biological relations, there is another relation that exists between mother and child that is an emotional connection. The feeling of being not able to bear the child or early demise of a child can make a strong psychological impact on the mother as reported in many cases.

### 7.3 Deceptive terminologies

MRT has been associated with a number of terms, some of which conveyed positive implications like “Mitochondrial gene therapy”, “Mitochondrial donation”, “Life-saving Treatment” (Ravitsky et al., 2015), “Narratives of Hope” (Herbrand and Dimond, 2017) while some others made negative impacts like “Three parent baby”, “Three-person baby”, “Three persons DNA” (Gonzalez-Santos et al., 2018), “Slippery Slope” (Carr, 2015), “Designer babies” (Kaur and Nagpal, 2017). As stated the term third parent name given to mitochondrial donor is highly misleading and non-sense (Dimond and Stephens, 2017). It is the nuclear DNA around which the whole concept of child's genetic identity and personality revolves as the former is the one to make a profound impact on the latter, not the mitochondrial DNA.

### 7.4 Germline intervention: a misconception

Critics of the technique are concerned about manipulation of the embryos considering it invalid morally which in actual, is a misconception. MRT is just like organ replacement *viz.* heart or kidney transplant or blood donation as culminated by the UK Department of health (Dimond, 2015). It involves the substantial replacement of defective mt-DNA with the healthy one without altering the sequence of genes inside it as in the case of genetic engineering (Pompie and Pompie, 2018) securing human asceticism.

### 7.5 Low chances of discrepancy

There are minimal chances of risks correlated with the mixing of prospective mother's mt-DNA and donor mt-DNA (Dolitsky and Saeur, 2019). The proofs about discrepancies are not reported yet. It is feasible to match the haplotypes of the two (Chinnery et al., 2014). There are little possibilities of mt-DNA alterations and is unlikely to be troublesome (Dimond, 2018).

### 7.6 Creation of healthy babies

MRTs are mistaken as curatives of mitochondrial disease. The objective of the approach is not to cure mitochondrial diseases rather to create genetically related healthy babies. It is wrong to equalize the term cure and creation (Rulli, 2016a, b). Indirectly it can minimize its prevalence by terminating the inheritance of mitochondrial disorders from diseased mother to her progenies (Gonzalez or Cavaliere, 2018). Thus, it is a beneficial approach according to the world's famed recite, “Prevention is better than cure”.

### 7.7 Limitations of other alternatives

Egg donation can lead to ovarian hyperstimulation syndrome and its genetic kinship cannot be achieved as the whole gamete (nuclear and mt-genome) is shared by a third person (Dimond, 2015). Surrogacy cannot be opted for mitochondrial disorder affected couple as it is the only womb shared by the third party and the child will possess the same mt-DNA as that of the mother. Prenatal diagnosis is unsuccessful in heteroplastic populations (Dimond, 2015). Pre-implantation Genetic Diagnosis (PGD) is appropriate for women with low levels of deficient mt-DNA (Pickett et al., 2019). It is unclear in the prediction of the disease due to "heteroplasm" and "genetic bottleneck" (Amato et al., 2014) and is inappropriate for homoplasmic populations (Rai et al., 2018). The drawbacks of these alternatives illustrate the importance of MRT for couples with mitochondrial diseases.

### 7.8 Lesbian couples

MRT also proves itself serviceable for lesbian couples because both the females in a lesbian couple can bestow their genetic portion to their child one being contributing nuclear genome and the other, the mitochondrial genome. There is another reproductive technique, permitting lesbian couples to have a child termed as ROPA (Reception of Oocyte from Partner). But the issue with ROPA is that the child is genetically related to one female only (Cavaliere and Gonzalez, 2018). So MRT should also be permitted to lesbian couples.

### 7.9 Social consent

Interestingly, the argument that the technique is socially inappropriate is supported by a handful of critics without knowing the approval of a wider segment of society to which the technique will eventually be applied. A survey was conducted by Englestad et al. (2016) to assess support for MRT. It disclosed that 95 percent of female carriers favored the implementation of the technique, while 52 percent of women considered it to be very important and 43 percent considered it to be somewhat important on the basis of a desire to have a genetic child. A further study of women suffering from mitochondrial diseases, after understanding all the risks associated, was conducted in the USA. The results of the survey show that 95% of women gave their approval to MRT (Pompie and Pompie, 2018). This demonstrates that if MRT is permitted to work, there would be widespread social acceptance.

## 8 Conclusion

MRT is surely a breakthrough in the world of medical science. But due to shortsighted disadvantages and misinterpretation of the title, the technique is clutched into the strictly prohibited laws of the government in various countries mistaking it to be a sort of “Germline Modification”. MRT seems to be the only option for patients affected with mitochondrial impairment desiring to have a genetic child which is neither immoral nor illicit. The purpose of this review is not to demean other reproductive approaches but to put off the blindfold claiming that MRT disturbs the sacrosanctity of humanity or disrespects cultural and social values. If permitted under appropriate regulatory laws, strict clinical researches, and carefully monitored conditions, the substantial risks can be minimized ensuring safe results and high standards of reproductive medicine.

## Declarations

### Author contribution statement

All authors listed have significantly contributed to the development and the writing of this article.

### Funding statement

This research did not receive any specific grant from funding agencies in the public, commercial, or not-for-profit sectors.

### Competing interest statement

The authors declare no conflict of interest.

### Additional information

No additional information is available for this paper.
